# Idiopathic 2:1 AV Block With Ventriculophasic Sinus Arrhythmia in a Young Male

**DOI:** 10.1016/j.jaccas.2026.108790

**Published:** 2026-07-29

**Authors:** Christopher Tortorici, Gustavo Duarte, Justin D. Mark, Roshan Wardak, Jose Muniz, Elias Collado

**Affiliations:** a Cleveland Clinic Florida, Weston, Florida, USA; b Holy Cross Health, Fort Lauderdale, Florida, USA

**Keywords:** bradycardia, cardiac magnetic resonance, electrophysiology, electrocardiogram, exercise

## Abstract

**Background:**

Second-degree atrioventricular (AV) block in young adults is rare and typically associated with structural, infectious, or genetic etiologies. Ventriculophasic sinus arrhythmia is classically associated with complete heart block and is rarely described in isolated 2:1 AV block.

**Case Summary:**

A 23-year-old male with no prior medical history presented to the clinic with influenza-like symptoms and was incidentally found to have marked bradycardia. Electrocardiogram revealed 2:1 AV Block with right bundle branch block, right-axis deviation, and ventriculophasic sinus arrhythmia. Comprehensive workup excluded structural and infiltrative disease. Exercise stress testing demonstrated transient 3:1 and 4:1 AV block, suggesting infranodal disease. A dual-chamber pacemaker was implanted with improved functional capacity.

**Discussion:**

This case highlights a rare presentation of idiopathic 2:1 AV block with ventriculophasic sinus arrhythmia and underscores the diagnostic value of exercise testing.

**Take-Home Message:**

Ventriculophasic sinus arrhythmia in 2:1 AV block may suggest intrinsic conduction system disease and supports early permanent pacing, even in minimally symptomatic patients.

## Visual Summary

**Figure:**
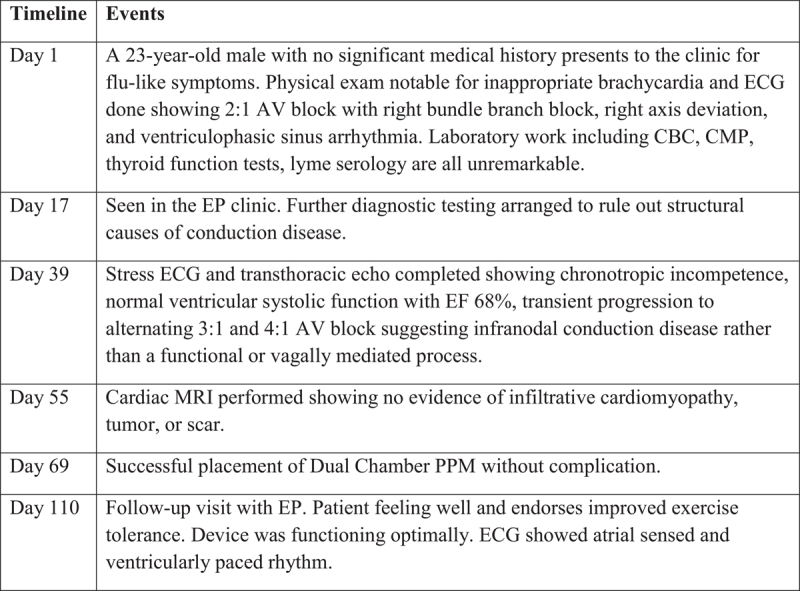

## History of Presentation

A 23-year-old male presented to the outpatient clinic with 1 day of influenza-like symptoms including rhinorrhea, sore throat, myalgias, headache, and fever. The patient denied any recent sick contacts. Vitals taken in the clinic showed a heart rate of 38 beats/min, oxygen saturation of 99% on room air, temperature of 100.5  F, and blood pressure of 121/48. Cardiac auscultation revealed a regular rhythm without murmurs, rubs, or gallops. The remainder of the physical exam was unremarkable. He denied chest pain, palpitations, dyspnea, syncope, presyncope, or lower-extremity edema. He reported lifelong mild exercise intolerance compared with peers but no prior medical evaluation. A rapid influenza test was positive during the visit. Given the degree of bradycardia in the setting of febrile illness, an electrocardiogram (ECG) was performed which demonstrated a 2:1 atrioventricular (AV) block with ventriculophasic sinus arrhythmia, right bundle branch block, and an unexplained right-axis deviation concerning for a possible left posterior fascicular block (Figure 1).

**Figure 1:**
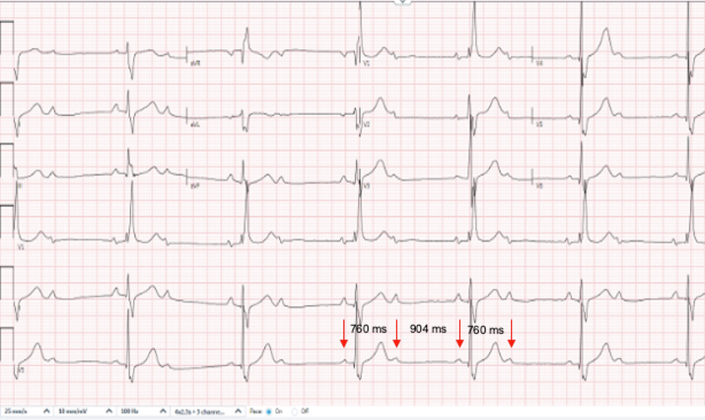
Initial Electrocardiogram Obtained in Clinic Demonstrating 2:1 Atrioventricular Block with Ventriculophasic Sinus Arrhythmia, Right Bundle Branch Block Morphology, and Right-Axis Deviation Concerning for a Left Posterior Fascicular Block Note the alternating P-P intervals are best appreciated in the rhythm strip (V3).

## Past Medical History

The patient had no known medical conditions and was not taking any prescription medications. He denied any history of tobacco use or illicit drug use. He reported occasional alcohol consumption. He exercised 3 to 4 times per week on average with light resistance training and walking. He used to play multiple sports recreationally in high school. He noted mild issues with exercise tolerance for most of his life including inability to keep up with his peers in aerobic activities. He had not sought prior medical evaluation, as his symptoms were mild and did not significantly impact daily functioning. Family history was negative for any cardiovascular disease, sudden death, or pacemaker implantation.

## Differential Diagnosis

The differential diagnoses included myocarditis, cardiac sarcoidosis, nonischemic cardiomyopathy, infectious etiologies of acquired AV block such as Lyme carditis, vagally mediated AV block, congenital AV Block, and congenital/inherited His-Purkinje system diseases such as Lenègre-Lev disease.

## Investigations

A thorough workup was completed after the patient's initial clinic visit. Laboratory testing with complete blood count, complete metabolic panel, and thyroid function tests were unremarkable. Lyme serology was negative. An exercise stress test with concurrent echocardiography and ECG demonstrated poor chronotropic response, achieving only 32% of the maximum predicted heart rate despite achieving 11.7 metabolic equivalents. During exercise, transient progression to 3:1 and 4:1 AV block was observed suggesting infranodal conduction disease (Figure 2). The patient remained asymptomatic, and no evidence of ischemia was detected. Transthoracic echocardiography showed preserved biventricular systolic function with mild left atrial enlargement. Cardiac magnetic resonance imaging demonstrated no evidence of infiltrative cardiomyopathy; normal T1 relaxation, no late gadolinium enhancement, or increased extracellular volume.

**Figure 2:**
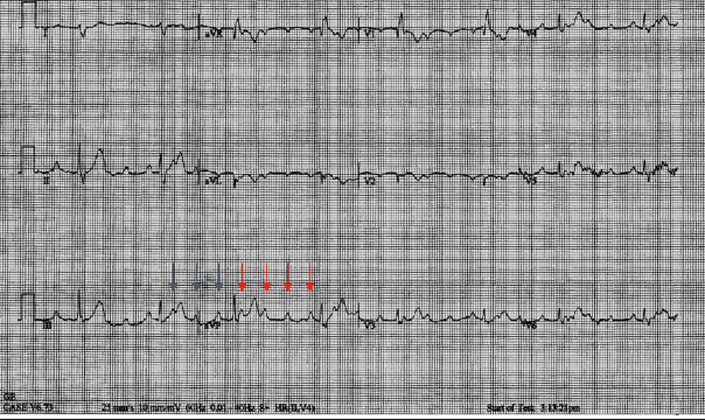
Electrocardiogram Obtained During Exercise Stress Testing at 3 minutes and 50 seconds Demonstrating Progression From 3:1 Atrioventricular Block to 4:1 Atrioventricular Block The nonconducted P waves during a 3:1 atrioventricular (AV) block are indicated by blue arrows. Subsequent progression to a 4:1 AV block is denoted by red arrows.

## Management

Given a persistent 2:1 AV block with exercise induced progression to a higher-degree block, permanent pacemaker placement was recommended. Conduction system pacing was initially attempted, including His-bundle pacing; however, selective His capture could not be achieved despite multiple attempts. The patient ultimately underwent implantation of a dual-chamber pacemaker with placement of a high septal right ventricular lead using a lumenless active-fixation 4F lead, achieving stable septal pacing with acceptable sensing and pacing thresholds (Figure 3). The device was programmed for dual-chamber rate-modulated pacing at 40 to 140 beats/min. Genetic testing was discussed; however, the patient deferred at this time.

**Figure 3:**
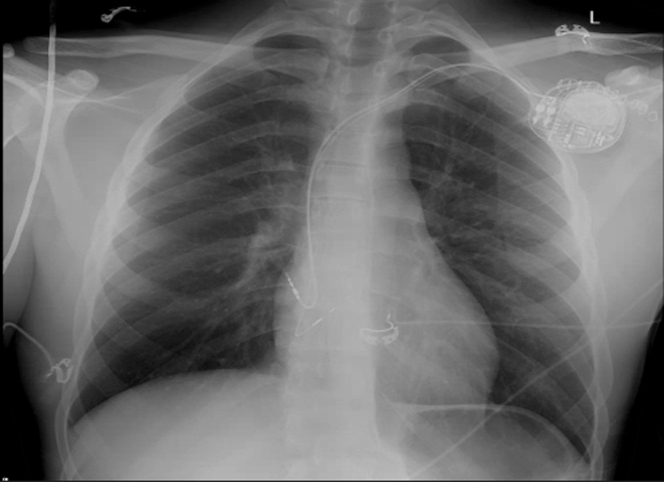
The Chest X-Ray Taken After the Procedure Shows Proper Placement of the Dual-Chamber Permanent Pacemaker

## Outcome and Follow-Up

At 2-month follow-up, the patient reported improved exercise tolerance. Pacemaker interrogation demonstrated appropriate device function with stable sensing and pacing thresholds and a predominant atrial-sensed, ventricularly paced rhythm (Figure 4). Although the patient was not pacemaker-dependent, his intrinsic PR interval remained markedly prolonged (>350 milliseconds), resulting in a high ventricular pacing burden despite adaptive AV delay programming. Longitudinal follow-up over 5 years has shown preserved left ventricular ejection fraction and stable cardiac chamber dimensions. The patient remains under routine electrophysiology surveillance without development of pacing-related cardiomyopathy or clinical deterioration.

**Figure 4:**
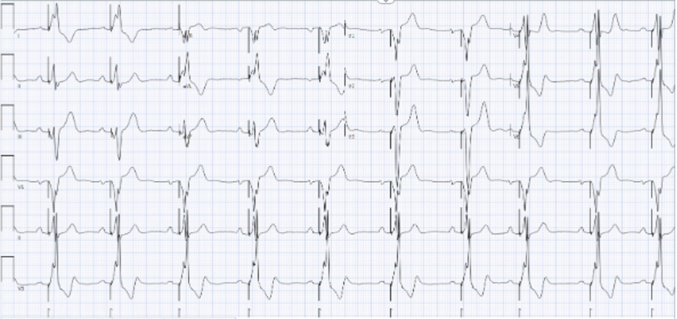
Postprocedural Electrocardiogram Demonstrating an Atrial Sensed and Ventricularly Paced Rhythm After Successful Placement of a Dual-Chamber Permanent Pacemaker

## Discussion

High-grade AV block in young adults is uncommon and most often associated with reversible or structural etiologies.^1^ When extensive evaluation fails to identify an underlying cause, idiopathic conduction system disease is considered.^1^^,^^2^ Ventriculophasic sinus arrhythmia is typically observed in the setting of complete AV block, where the atrial and ventricular rhythms are dissociated. It is characterized by a shortening of the P-P interval when a QRS complex is interposed between two P waves.^3^, ^4^, ^5^ Although the mechanism is poorly understood, it is proposed to be secondary to baroreceptor activation in the carotid sinus resulting from ventricular contraction on the conducted beats, leading to a shortening of the subsequent P-P interval. Another proposed mechanism involves changes in autonomic tone of the sinus node in response to ventricular systole, which transiently accelerates sinus node discharge via pulsatile blood flow through the sinus nodal artery creating increased stretch and activation.^3^, ^4^, ^5^ This phenomenon is rarely reported in isolated 2:1 AV blocks. Published case reports describing this phenomenon involve predominantly older patients often in the setting of age-related degenerative conduction system disease. A similar ECG finding has been described in a young adult with reversible etiology, specifically Lyme Carditis.^6^ However, to our knowledge, this represents one of the youngest reported cases of idiopathic ventriculophasic sinus arrhythmia with a 2:1 AV Block. The progression of this patient's AV block during exercise supports infranodal disease rather than a functional or vagally mediated mechanism.^7^^,^^8^ This finding has important management implications, as current guideline recommendations support permanent pacing in patients with high-grade AV block and evidence of infranodal involvement, even in the absence of overt symptoms.^1^ Although conduction system pacing is increasingly favored in young patients to minimize long-term ventricular dys-synchrony, its adoption was limited at the time of this patient's presentation in early 2018. Left bundle branch area pacing has since emerged as an additional conduction system pacing strategy, although it had not yet been widely adopted at the time of this case. Attempts at His-bundle pacing were made during the procedure using multiple specialized delivery catheters; however, a discrete His deflection could not be reliably identified, and His capture could not be achieved. Consequently, precise electrophysiologic localization of the level of block during the procedure was limited, although the exercise-induced progression to higher-grade AV block remained clinically concerning for infranodal disease. A high-septal right ventricular pacing strategy was subsequently pursued with successful capture.

Current literature suggests that young patients with an idiopathic AV block have an increased long-term risk of adverse cardiovascular outcomes, including heart failure, ventricular arrhythmias, and mortality, compared with age-matched controls.^1^^,^^2^ These observations highlight the importance of early intervention, close surveillance, and consideration of genetic evaluation in selected patients. In this case, genetic counseling and testing were discussed but deferred by the patient. Current guidelines do not endorse routine genetic testing in all patients with conduction disease; however, testing may be considered in young patients with idiopathic or progressive disease per American College of Cardiology/American Heart Association/Heart Rhythm Society guidelines (class IIb recommendation).^9^ Contemporary European Society of Cardiology guidance recommends consideration of targeted genetic testing in young patients with otherwise unexplained progressive conduction disease, particularly when the diagnosis was made at an early age or when there is suspicion for inherited conduction system disorders.^10^ Notably, this case occurred in 2018 prior to publication of these European Society of Cardiology recommendations. In retrospect, such testing would be reasonable and remains an important consideration during longitudinal follow-up.

## Conclusions

This case illustrates a rare presentation of an idiopathic 2:1 AV block with ventriculophasic sinus arrhythmia in a young adult. Recognition of ventriculophasic sinus arrhythmia and exercise-induced progression of an AV block may provide important clues to advanced conduction system disease and support timely permanent pacing to mitigate potential long-term cardiovascular risk, even in minimally symptomatic patients.

## Funding Support and Author Disclosures

The authors have reported that they have no relationships relevant to the contents of this paper to disclose.

**Take-Home Messages**

• Idiopathic second-degree AV block in young patients warrants thorough evaluation and may justify early permanent pacing, even in minimally symptomatic individuals.
• Ventriculophasic sinus arrhythmia in a partial AV block is rare and may signal more advanced conduction system disease.
• Exercise testing can aid in risk stratification and guide management decisions in young patients with a second-degree AV block.

**Visual Summary:** Clinical Timeline of Diagnostic Evaluation and Management

Timeline	Events
Day 1	A 23-y-old male with no significant medical history presents to the clinic for flu-like symptoms. Physical exam notable for inappropriate bradycardia and ECG done showing 2:1 AV block with right bundle branch block, right-axis deviation, and ventriculophasic sinus arrhythmia. Laboratory work including CBC, CMP, thyroid function tests, lyme serology are all unremarkable.
Day 17	Seen in the EP clinic. Further diagnostic testing arranged to rule out structural causes of conduction disease.
Day 39	Stress ECG and transthoracic echo completed showing chronotropic incompetence, normal ventricular systolic function with EF 68%, transient progression to alternating 3:1 and 4:1 AV block suggesting infranodal conduction disease rather than a functional or vagally mediated process.
Day 55	Cardiac MRI performed showing no evidence of infiltrative cardiomyopathy, tumor, or scar.
Day 69	Successful placement of dual-chamber PPM without complication.
Day 110	Follow-up visit with EP. Patient feeling well and endorses improved exercise tolerance. Device was functioning optimally. ECG showed atrial sensed and ventricularly paced rhythm.

AV = atrioventricular; CBC = complete blood count; CMP = comprehensive metabolic panel; ECG = electrocardiogram; EF = ejection fraction; EP = electrophysiology; MRI = magnetic resonance imaging; PPM = permanent pacemaker.
